# Comprehensive study upon physicochemical properties of bio-ZnO NCs

**DOI:** 10.1038/s41598-023-27564-w

**Published:** 2023-01-11

**Authors:** Anna Król-Górniak, Viorica Railean, Paweł Pomastowski, Tomasz Płociński, Michał Gloc, Renata Dobrucka, Krzysztof Jan Kurzydłowski, Bogusław Buszewski

**Affiliations:** 1 Department of Environmental Chemistry and Bioanalysis, Faculty of Chemistry, Nicolaus Copernicus University in Toruń, Gagarina 7, 87-100 Toruń, Poland; 2 Centre for Modern Interdisciplinary Technologies, Nicolaus Copernicus University in Toruń, Wileńska 4, 87-100 Toruń, Poland; 3 Department of Infectious, Invasive Diseases and Veterinary Administration, Institute of Veterinary Medicine, Nicolaus Copernicus University in Torun, Gagarina 7, 87-100 Toruń, Poland; 4 Faculty of Materials Science and Engineering Warsaw, University of Technology, ul. Wołoska 141, 02-507 Warsaw, Poland; 5 Department of Non-Food Products Quality and Packaging Development, Institute of Quality Science, Poznań University of Economics and Business, al. Niepodległości 10, 61-875 Poznań, Poland; 6 Faculty of Mechanical Engineering, Bialystok University of Technology, ul. Wiejska 45C, 15-351 Bialystok, Poland

**Keywords:** Biochemistry, Biotechnology, Chemical biology

## Abstract

In this study, for the first time, the comparison of commercially available chemical ZnO NCs and bio-ZnO NCs produced extracellularly by two different probiotic isolates (*Latilactobacillus curvatus* MEVP1 [OM736187] and *Limosilactobacillus fermentum* MEVP2 [OM736188]) were performed. All types of ZnO formulations were characterized by comprehensive interdisciplinary approach including various instrumental techniques in order to obtain nanocomposites with suitable properties for further applications, i.e. biomedical. Based on the X- ray diffraction analysis results, all tested nanoparticles exhibited the wurtzite structure with an average crystalline size distribution of 21.1 nm (CHEM_ZnO NCs), 13.2 nm (1C_ZnO NCs) and 12.9 nm (4a_ZnO NCs). The microscopy approach with use of broad range of detectors (SE, BF, HAADF) revealed the core–shell structure of bio-ZnO NCs, compared to the chemical one. The nanoparticles core of 1C and 4a_ZnO NCs are coated by the specific organic deposit coming from the metabolites produced by two probiotic strains, *L. fermentum* and *L. curvatus*. Vibrational infrared spectroscopy, photoluminescence (PL) and mass spectrometry (LDI-TOF-MS) have been used to monitor the ZnO NCs surface chemistry and allowed for better description of bio-NCs organic coating composition (amino acids residues). The characterized ZnO formulations were then assessed for their photocatalytic properties against methylene blue (MB). Both types of bio-ZnO NCs exhibited good photocatalytic activity, however, the effect of CHEM_ZnO NCs was more potent than bio-ZnO NCs. Finally, the colloidal stability of the tested nanoparticles were investigated based on the zeta potential (ZP) and hydrodynamic diameter measurements in dependence of the nanocomposites concentration and investigation time. During the biosynthesis of nano-ZnO, the increment of pH from 5.7 to around 8 were observed which suggested possible contribution of zinc aquacomplexes and carboxyl-rich compounds resulted in conversion of zinc tetrahydroxy ion complex to ZnO NCs. Overall results in present study suggest that used accessible source such us probiotic strains, *L. fermentum* and *L. curvatus*, for extracellular bio-ZnO NCs synthesis are of high interest. What is important, no significant differences between organic deposit (e.g. metabolites) produced by tested strains were noticed—both of them allowed to form the nanoparticles with natural origin coating. In comparison to chemical ZnO NCs, those synthetized via microbiological route are promising material with further biological potential once have shown high stability during 7 days.

## Introduction

Recently, the biological synthesis of nanoparticles (NCs) seem to be a matter of concern as an attractive alternative for the commonly applied chemical and physical approaches^1–3^. Over the few last years, the number of publication focused on the bio-NCs has increased significantly and it is still rising. In particular, zinc oxide nanoparticles (ZnO NCs) have attracted scientific interest, due to their broad applications from sensors, electronic devices^4,5^ to antimicrobial agents or drug delivery^6,7^. Many research groups have reported the possibility of bacteria, fungi and plant extracts usage for bio-ZnO nanomaterials production^8–16^. The main object of this approaches was to develop eco-friendly synthesis protocol, search for newer and newer biological sources (plant extracts or bacteria strains), and in the consequence, to obtain nanoparticles with unique properties (e.g. antimicrobial activity and low cytotoxicity). Some reports, including our works, have described the effective bio-ZnO NCs microbial synthesis using lactic acid bacteria (LAB) strains^10,13–16^. LABs have the GRAS status (*generally recognized as safe*) and they are easily accessible from the dairy products (milk, yoghurt or whey). Moreover, LAB’s antimicrobial properties has been proved by many authors^17,18^. Therefore, it might be assumed that this type of microorganism is promising material for nanomaterials production. El-Sayed et al^14^ have performed the intracellular synthesis with the use of *Lactobacillus gasseri* strain and then applied as an yoghurt additives. Yusof with colleagues have proposed both, intra-^16^ and extracellular^15^, ZnO NCs synthesis by *Lactobacillus plantarum* strain TA4 isolated from local fermented food. The work of Pomastowski et al.^13^ described the extracellular ZnO synthesis by *Lactobacillus paracasei* LC20 isolated from sweet whey. The nanomaterials obtained in cited study exhibited good antimicrobial activity toward pathogenic strain. Most of the published papers underlined the advantages of biosynthesis toward the chemical approach. Biosynthesis it is reported to involve a lower use of expensive, toxic reagents and show higher efficiency. Nevertheless, the variability of biological sources used for bio-ZnO NCs preparation might be a limiting factor to standardize the synthesis conditions. Depending on the type of biomolecules present in the e.g. bacterial supernatant, we are able to obtain nanomaterials with different size, morphology and stability. Also, such factors as pH, temperature, time of bacteria cultivation, or precursor salt concentration will influence the further properties of bio-NCs. Kordy et al.^19^ have shown that the size of ZnO NCs produced extracellularly by *Alkalibacillus* sp. W7 strongly depends on factors such as pH, precursor concentration and temperature of incubation. They have observed that the smallest nanoparticles (around 21 nm) were produced at pH 8**,** with 8 mM ZnSO_4_.7H_2_O at 30 °C^19^. Rose et al.^20^ have optimized the conditions of extracellular silver nanoparticles (Ag NCs) synthesis using supernatant after the *Penicillium oxalicum* cultivation. In addition to factors such as pH, synthesis temperature and AgNO_3_ concentration, they also took into account the presence of specific enzymes in the yeast supernatant. Their data have pointed out the crucial role of the nitrate reductase for the maximum production of Ag NCs.

Simultaneously, commercially available chemical nanoparticles require rigorous quality standards including size and stability. Then, several types of NCs surface modifications are applied for their stabilization^21–23^. Chemicals including polyethylene glycols (PEGs), sodium citrate^24^, butyl acetate^25^, aminopropyltriethoxysilane (APTES)^26^ and many others are commonly used for capping of nanomaterials. In the case of biological synthesis, it is possible to obtain the NCs with the specific organic coating onto NCs core. Al-Kordy et al.^19^ have proved that the metabolites of *Alkalibacillus* sp.W7 strain acted as viable capping and stabilizing agents for ZnO NCs. Moreover, our latest papers have shown that extracellularly synthetized nanoparticles (Ag and ZnO NCs) are naturally stabilized by the specific organic deposit on their surface, coming from the bacterial metabolites^13,27^. However, there is limited number of works comparing the chemical and biological approaches in the context of the nanoparticles physicochemical characterization, e.g. morphology, thermal and colloidal stability or optical properties. The conditions during the chemical synthesis are easier to control and pre-determined, while the application of biological system caused the possible variation in biomolecules (metabolites, proteins, enzymes, etc.) content. Consequently, biological synthesis of NCs is still under many doubts and questions as far as critical comparisons between NCs synthesized through different routes are not performed.

The motivation to undertake this research subject was the interest of ZnO NCs biological synthesis NCs as an environmentally friendly and easy-to-handle method to obtain complex structures containing the zinc oxide core and specific organic deposit on the surface. The presence of bacterial metabolites residues around the nanoparticles core lead to design a novel and safe antimicrobial agent for treatment of bacterial infections. The outcomes of our previous studies have confirmed the ability of bio-ZnO NCs with surface organic deposit to act as a promising antiseptic against clinically relevant and antibiotic-resistant pathogens. However, taking into consideration guidelines of nano-ingredients safety assessment, we have decided to perform the comprehensive evaluation of bio-ZnO NCs physicochemical properties in comparison to commonly used and chemically synthetized zinc oxide nanomaterials. For that reason, in this paper, the commercially available ZnO NCs dispersed in butyl acetate and bio-NCs synthetized extracellularly by two probiotic strains (*L. fermentum* and *L. curvatus*) isolated from the milk were compared. All types of nanoparticles were subjected to microscopic (electron microscopy; atomic force microscopy), spectroscopic (Fourier transform infrared spectroscopy) and spectrometric (laser desorption/ionization with time of flight analyzer mass spectrometry, photoluminescence) examination. Additionally, the thermal and colloidal stability were investigated. The one of questions addressed in this paper is about the impact of ZnO NCs synthesis type on their further physicochemical features. Next, how the concentration of nanoparticles may influence their stability, size, optical properties and presence of organic surface coating. Another issue that needs to be considered is the impact of time on NCs colloidal stability. For this purpose, the zeta potential (ZP), size (DLS) and photoluminescence (PL) measurements of all ZnO NCs were performed as function of time or/and NCs concentration.

## Experimental section

### Isolation and identification of bacterial strain

The *Limosilactobacillus fermentum* MEVP2 [OM736188] (4a) and *Latilactobacillus curvatus MEVP1* [*OM736187*] (1C) were isolated from milk (Dairy Cooperative in Drzycim, Poland) according to the previously published protocol^27^. The molecular identification of bacterial strain was performed by 16S rDNA PCR method using universal 27 F and 1492R primers according to the method described in our work previously^10^. Additionally, the MALDI-TOF-MS identification by the ultrafleXtreme mass spectrometer (Bruker Daltonics, Hamburg, Germany) using formic acid-acetonitrile extraction and BioTyper identification. Finally, the isolated strains were deposited in the Polish Collection of Microorganisms (PCM) under deposit no. B/00399 and B/00400 for *L. curvatus* and *L. fermentum*, respectively.

### Extracellular synthesis of ZnO NCs

The probiotic strains isolated from milk were cultivated on Müller–Hinton broth (MH, Sigma-Aldrich, St. Louis, MO, USA) and incubated at 27 °C for 48 h. Both strains were inoculated and cultivated separately. After the incubation, the cultures were centrifuged at 12,000 rpm for 20 min and the collected cell-free supernatant were used for further ZnO NCs biosynthesis. Before the biosynthesis, the supernatant were diluted with distilled water in 1:1 proportion (*v*:*v*). The zinc nitrate (Zn(NO_3_)_2_) at 0.1 g/mL concentration was chosen as a precursor and added to the diluted supernatant under continuous magnetic stirring. Then, the synthesis was carried out at 60 °C for 3 h. After this time, the mixture was centrifuged (10,000 rpm, 15 min) and the obtained supernatant was transferred to a new container and heated at 120 °C until a dried precipitate appeared. The collected ZnO powder was washed with distilled water (10 times) by centrifugation (10 min, 7000 rpm) and then, all unreacted ions as well as low molecular weight metabolites were removed by 5-day dialysis (3 kDa cutoff, Spectrum Lab, Thermo Fisher Scientific, Vantaa, Finland). The pH value was monitored at each step of biosynthesis. In order to prove the effect of the biological method the same protocol was followed without bacterial strain. As a control, the medium broth instead of bacterial supernatant was used.

The further physicochemical characterization of obtained biological ZnO NCs (1C and 4a_ZnO NCs) were performed in comparison to the chemical ZnO NCs obtained from Sigma-Aldrich were used (CHEM_ZnO NCs).

### Physicochemical characterization of final product (ZnO NCs)

#### X-ray diffraction

The crystalline structure of the chemical (CHEM_ZnO NCs) and biological (1C_ and 4a_ZnO NCs) nanoparticles, were investigated by X-ray diffraction method. For this purpose, the X’Pert Pro Analytical diffractometer (Phillips, Erlangen, Germany) with CuKα (λ = 1.54056 Å) radiation source and Ni filter was applied. The XRD diffraction patterns were analyzed by XRD Malvern Panalytical software (version 1.5a, Almelo, The Netherlands). Then, the crystallite size of all tested ZnO NCS was calculated based on the Scherrer equation:

1 $$d = \frac{k\lambda }{{\beta \cos \theta }}$$

where λ is the wavelength of the X-ray; β, FWHM width of the diffraction peak; θ, diffraction angle; and k, constant according to the Patterson^28^.

#### Imaging techniques

For imaging of all tested ZnO NCs types, the various microscopic approaches were applied, including:

- Scanning electron microscopy (SEM, LEO 1430 VP, Leo Electron Microscopy Ltd, Cambridge, United Kingdom) coupled with an energy dispersive X-ray (EDX) detector (XFlash 4010, Bruker AXS, Bremen, Germany),
- Transmission electron microscopy (TEM, FEI Tecnai F20 X-Twintool, FEI Europe, Frankfurt/Main, Germany),
- Scanning transmission electron microscopy (STEM, HD2700 model, Hitachi High Technologies, Japan)
- Atomic force microscopy (AFM, Veeco SPM (Digital Instrument) equipped with NanoScope IIIa controller and Quadrex, MultiMode microscope, E type scanner with maximum scanning area 10 × 10 × 2.5 µm). A thin film of ZnO NCs suspension was deposited on silica glass plate via dropping method and then dried at room temperature for 4 h. The samples have been imaged in tapping mode using HN-NC cantilevers with a resonance frequency of 120 kHz; the scan speed was set to 1.97 Hz. The obtained data have been operated via NanoScope Analysis software V1.4 and the depth histogram row data (size distribution) were derived automatically by the instrument-equipped software and processed by ORIGIN Pro/2016 software.

Accordingly, the SEM analysis were performed with the secondary electrons (SE) mode. The TEM images were collected with the bright field (BF) and dark field (DF) mode. Additionally, the selected area electron diffraction (SAED) pattern as well as the energy dispersive X-ray (EDX) spectra were recorded. Finally, the STEM approach were used with three different detectors: High Angle Annular Dark Field detector (HAADF), fast Fourier transform (FFT), SE and BF.

#### Fourier transform infrared (FT-IR) spectroscopy

In order to describe the organic surface coating of all tested ZnO NCs including active functional groups, the Bruker FTIR-ATR (Billerica, Massachusetts, USA)spectroscopy was carried out in the range of υ = 400–4000 cm^−1^ (Spectrum 2000; The registered data were proceeded with OPUS 7.5 software (Bruker Daltonics, Hamburg, Germany).

#### Laser desorption/ionization with time of flight analyzer mass spectrometry (LDI-TOF-MS)

The composition of organic surface coating of all tested ZnO NCs were investigated by the laser desorption/ionization with time of flight analyzer mass spectrometry. The LDI-TOF-MS analysis was carry out using ultrafleXtreme mass spectrometer (Bruker Daltonics, Hamburg, Germany) without any matrix. The all types of ZnO NCs were prepared according to the previously described protocol^13^. For the proper calibration, the solutions of protein Calibration Standards I and Peptide Calibration Standard Bruker Daltoniks, Bremen, Germany) were used. The registered molecular fingerprint spectra of CHEM, 1C and 4a_ZnO NCs were further analyzed with the FlexControl and FlexAnalysis (Bruker Daltonics, Hamburg, Germany) software. The LDI-TOF-MS analysis were carried out for the 62.5, 250 and 500 µg/mL concentration of all tested nanoparticles.

#### Photoluminescence (PL) of ZnO NCs

The chemical and biological ZnO NCs were subjected to the analysis of nanoparticles photoluminescence with a JASCO FP-8300 spectrofluorometer (JASCO Europe, Cremella, Italy). The photoluminescence (PL) spectra for zinc oxide signal and organic coating of nanoparticles were recorded with 5 nm wavelength interval in the 210–735 nm range. As a control, the water sample was used. All experiment were conducted in triplicate.

#### Photocatalytic activity of ZnO NCs

All tested ZnO NCs (at 1000 µg/mL concentration) were implemented as a photocatalysts toward methylene blue (MB) degradation under sunlight irradiation. The photocatalytic activity of CHEM, 1C and 4a_ZnO NCs were evaluated according to the previously described protocol^29^. The process were carried out in time intervals (1, 3, 6, 8, 12 and 48 h) and in triplicate. The MB concentrations were monitored at λ = 665 nm during using Varioskan TM LUX multimode microplate reader (Thermo Fisher Scientific, Waltham, MA, USA).

#### Colloidal stability and size distribution of ZnO NCs

The colloidal stability of CHEM, 1C and 4a_ZnO NCs was determined by zeta potential (ZP) and hydrodynamic diameter measurements. The nanoparticles zeta potential and size distributions were determined with Zetasizer Nano Series (Malvern Instruments, Malvern, Great Britain) for samples at 62.5, 125, 250, 500 and 1000 µg/mL. Moreover, the impact of time (1, 2, 4 and 7 days of investigation) was included. Before the analysis, the samples were mixed on the vortex (Vortex Genie 2; IKA® Poland) for 5 min and sonicated for 10 min using the ultrasonic cleaner (USC THD model with 45 kHz ultrasonic frequency and degassing function, VWR International, Poland). For the hydrodynamic diameter and zeta potential measurements, the YV Grade cuvette and Folded Capillary Zeta Cells (Malvern Panalytical, Great Britain) were applied, respectively. All measurements were performed in triplicate. The obtained experimental data were calculated as an average value and presented with a standard deviation.

#### Thermogravimetric analysis (TG-DTA)

Thermal analysis of the final product of synthesis (ZnO NCs) was carried out by using TA Instruments type SDT 2960 (Artisan Technology, Champaign, IL, USA) in the range of 0–1000 °C with an air flow rate of 100 mL/min and heating rate of 10 °C/min. The collected data were processed by TGA-DTA thermal analysis software (version V5 7.0, TA Instruments, New Castle, DE, USA). All measurements were carried out in triplicate.

## Results and discussion

### X-ray diffraction (XRD)

**Figure 1:**
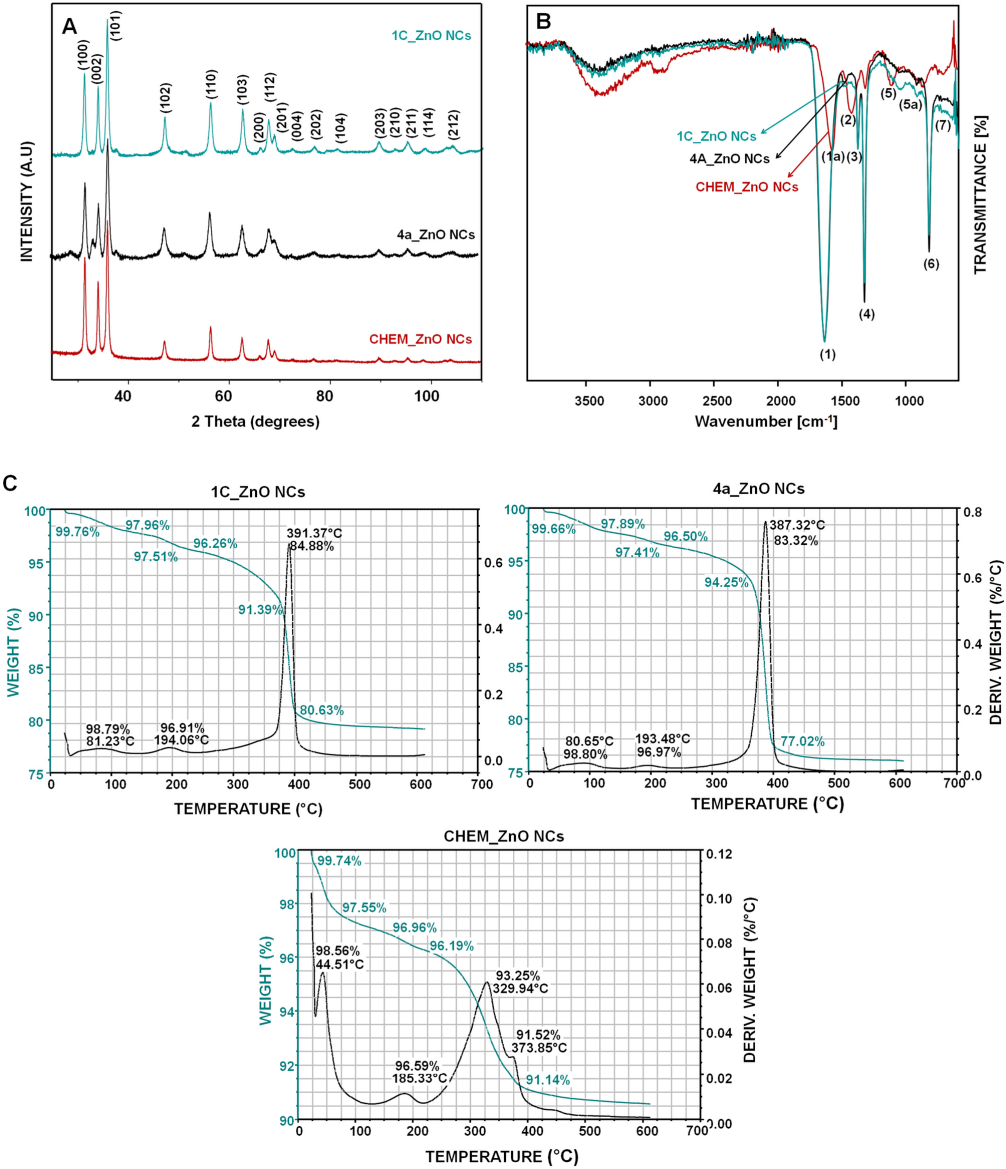
Physicochemical characterization of 1C, 4a and CHEM_ZnO NCs. XRD pattern (**A**); the FT-IR spectra (**B**) and the TGA/DTA pattern (**C**).

The crystallinity, purity, and phase identification of all tested ZnO NCs was determined by X-ray diffraction (XRD) method. As shown in the Fig. 1A, the CHEM_ZnONCs as well as the 4a and 1C_ZnO NCs showed the diffraction peak at 31.84, 34.54, 36.34, 47.63, 56.67, 62.93, 66.38, 67.91, 69.05, 72.14, 76.94, 81.46, 89.57, 92.74, 95.25, 98.63 and 102.92 corresponding to (100), (002), (101), (102), (110), (103), (200), (112), (201), (004), (202), (104), (203), (210), (211), (114) and (212) Bragg reflections, respectively^30^. Both chemical and biological synthesized ZnO NCs demonstrated similar crystallinity fitting into the wurtzite structure of zinc oxide^30^. The ZnO hexagonal wurtzite structure is characterized by following lattice parameters a = 0.325 nm and c = 0.521 nm and with three primary crystal surfaces—{1010}, {1120} and {0001}^31,32^. Among the dominant facets, the Zn- and O-faced {0001} surfaces are polar whereas the {1010} and {1120} surfaces are non-polar^32,33^.

In the case of biologically synthesized ZnO NCs some additional peak at 59.42° was observed. Based on literature, it might be assigned to the intermediate products of bio-ZnO NCs synthesis, such as zinc hydroxynitrate (Zn_5_(OH)_8_(NO_3_)_2_(H_2_O)_2_)^34,35^. What is interesting, no significant differences in the peaks intensity between the 1C and 4a_ZnONCs were observed. However, it should be noticed that 1C and 4a_ZnO NCs displayed broader diffraction peaks—according to the Scherrer Eq. (1) it indicates the smaller crystalline size in comparison to CHEM_ZnONCs (as shown in the Table 1).

**Table 1:** Average crystallite size of ZnO NCs calculated from the Scherrer’s equation.

Type of ZnO NCs	Crystalline size
1C_ZnONCs	13.2 ± 2.1 nm
4a_ZnONCs	12.9 ± 2.9 nm
CHEM_ZnONCs	21.1 ± 1.4 nm

Moreover, Nithya et al.^36^ have observed similar dependence—the chemically synthetized ZnO NCs were found to be at 65 nm while the bio-ZnO NCs prepared with the *C. halicacabum* leaf extract had the crystallite size around 42 nm. Haque et al.^37^ have compared the crystallite size of ZnO NCs prepared by sol–gel and biological (plant extract) methods. Both types of nanoparticles have demonstrated a wurtzite hexagonal structure, but the bio-ZnO NCs exhibited smaller crystallites (around 26 nm) than that chemical ones (33 nm). The differences between the crystallite size of tested nanoparticles might be related with the synthesis conditions. Referring to the literature^38–40^, the increase of the synthesis temperature resulted in the higher crystallite size. It is well known that chemical synthesis is performed in the high temperatures (up to 600 °C)^41–44^, whereas the designed in this paper biological synthesis procedure require the use maximum 110 °C. Another important factor possibly influencing the ZnO NCs crystallite size is the organic coating. Khanna with colleagues^45^ have investigated the effect of silica coating on the CuFe_2_O_4_ NCs physicochemical properties.

The data from the XRD analysis have indicated the smaller crystallite size (3 nm) of silica coated nanoparticles, in comparison with the uncoated NCs (7 nm). El-Nahhal et al.^46^ have observed the smaller mean crystallite size for chemically obtained ZnO NCs after their stabilization by different surfactants. Nevertheless, Anders et al.^47^ have observed that the binding the fetal bovine serum (FBS) proteins on ZnO NCs surface did not affect their crystallite size. In the case of presented study, it can be postulated that specific organic deposit coming from the bacterial metabolites in supernatant used for biosynthesis is crucial factor determining the crystallite size of bio-ZnO NCs. In our previous work^13^, the extracellular synthesis by *L. paracasei* LC20 resulted in the nanoparticles with organic deposit at around 14 nm crystallite size.

The presence of zinc oxide in the tested samples were also indirectly confirmed by the vibrational infrared spectroscopy. The signal registered at 525 cm^−1^ (7) in the FTIR-ATR spectra indicate the chemical bonding between zinc and oxygen suggesting the formation of ZnO nanoparticles by the both, chemical and biological, methods (Fig. 1B). The characterization of NCs surface coating registered in the ν = 400–4000 cm^−1^ range is discussed in the “Organic surface deposit (FTIR-ATR, LDI-MS)” section.

Thermal behavior of CHEM, 1C and 4a_ZnO NCs were studied using thermogravimetric (TGA) and differential thermal analysis (DTA) in the range of 0–700 °C, as shown in the Fig. 1C. The TGA/DTA analysis revealed that the chemical and bio-ZnO NCs loose about 4% of its weight at around 200 °C which might be related with the evaporation of residual water. Above 300 °C, the final weight loss for all ZnO NCs is observed. In the case of CHEM_ZnO NCs, two DTA peaks were observed—the first one at 329.94 °C (6.75% weight loss) and the second at 373.85 °C (8.48% weight loss) (Fig. 1C). On the other hand, bio-ZnO NCs exhibited only one DTA peak (391.37 °C; 15.12% weight loss and 387.32 °C; 16.68% weight loss for the 1C and 4a_ZnO NCs, respectively) (Fig. 1C). The observed thermal change in the range of 300–400 °C is characteristic for the final ZnO NCs crystallization during the heating process, which has been confirmed by X-ray diffraction. No additional weight loss was observed above 450 °C, which is related to wurtzite-type phase stability up to 700 °C^48^. Furthermore, the observed major weight loss might be also associated with the combustion of organic residues that were part of the butyl acetate dispersion (CHEM_ZnO NCs) or organic surface deposit (1C and 4a_ZnO NCs)^49^. Li et al.^44^ have observed similar effect—around 360 °C the decomposition of PEG-400 stabilizer at chemical ZnO NCs surface have occurred. The obtained data revealed a slightly lower thermal stability of bio-ZnO NCs compared to chemical and commercially available ones. What is more, TG/DTA approach provide insight into the nanoparticles structural composition (organic coating) which was broader investigated by spectroscopic and spectrometric methods (next section).

### Electron microscopy

Microscopic analysis at atomic resolution is often applied to provide deep insight into the structure and chemical composition of nanomaterials. Depending on the type of electron microscope and used detector, we are able to collect different signals and perform a comprehensive analysis. For instance, the bright field (BF) and high angle annular dark field (HAADF) scanning transmission electron microscopy (STEM) are usually chosen to describe the nanoparticles morphology. At the same time, energy dispersive X-ray spectroscopy (EDX) detector might be used for the sample elemental distribution analysis. In our work, various types of microscopic detectors, including BF, SE, HAADF and FFT let to the broader description of chemical and biological ZnO NCs core and shell structure.

**Figure 2:**
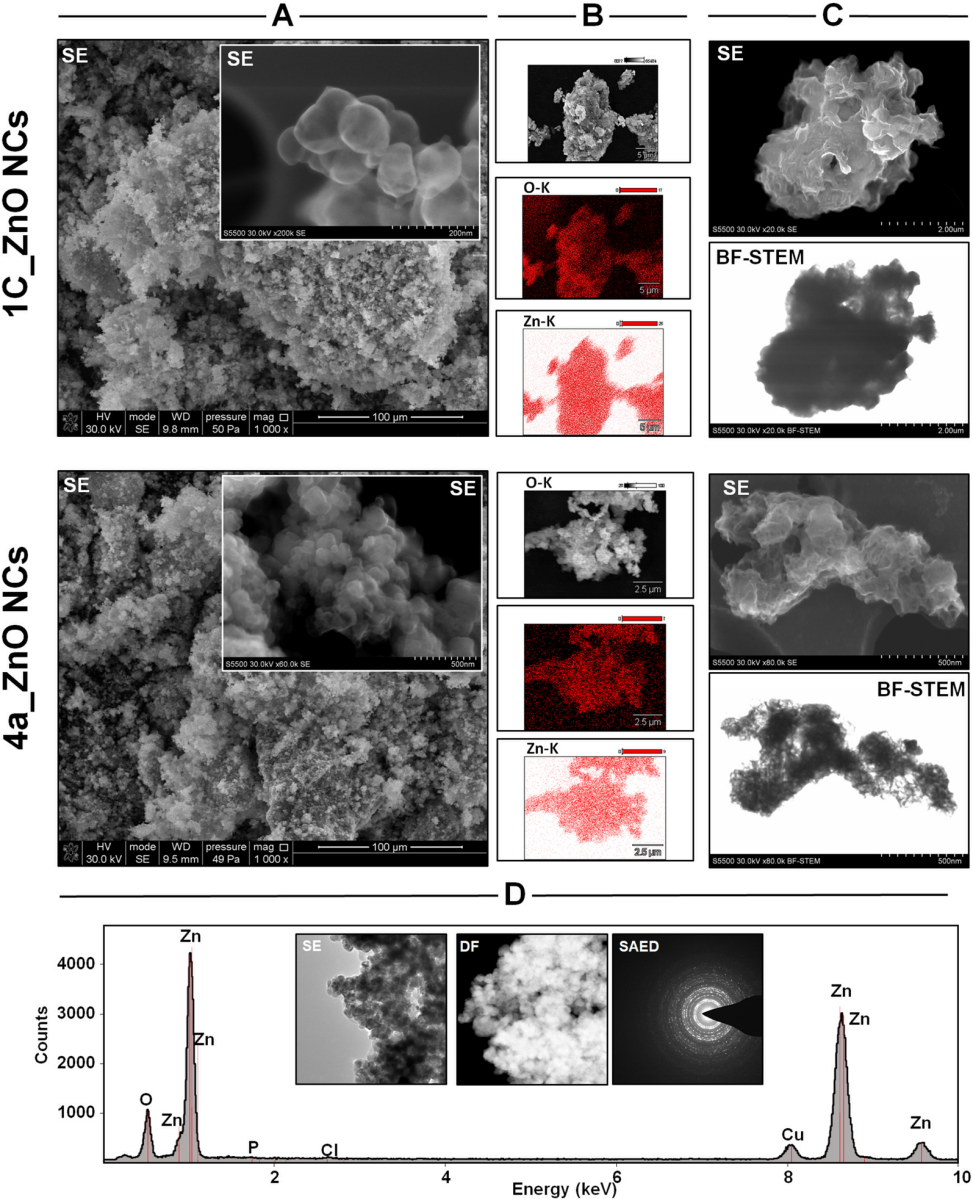
The electron micrographs for the 1C and 4a_ZnO NCs; SEM images in SE mode (**A**), SEM mapping (**B**), STEM images in SE and BF modes (**C**) and TEM images in BF, DF, SAED modes with EDX detector (**D**).

The Fig. 2A and B presents the scanning electron microscopy (SEM) images of 1C and 4a_ZnO NCs in the secondary electron (SE) mode with the mapping of biologically synthetized nanoparticles. On the Fig. 2C, the scanning transmission electron microscopy (STEM) images in SE and bright field (BF-STEM) work modes are shown. In turn, on the Fig. 2D, the transmission electron micrographs (TEM) are presented, together with the selected area of diffraction pattern (SAED) and energy dispersive X-ray (EDX) detection.

Comparing two detector modes, SE and BF-STEM, the complementary information about biological nanoparticles is provided. The SE mode commonly used in the SEM microscopy allows for description of the surface topography and size of tested materials^50,51^. On the other hand, the BF-STEM presents the image contrast for objects compared to the matrix^52,53^. Generally, STEM microscopy operate with a scanning electron probe, comparable to SEM. However, the STEM signals originate from electrons transmitted through a thin specimen. Therefore, the BF-STEM images give the information about the NCs crystallographic structure and mass-thickness rather than about the surface morphology^54^. According to the Fig. 2A and C (SE mode), both types of bio-ZnO NCs were characterized by similar surface morphology—they are rather spherical in shape. Based on the literature, in the BF-STEM mode (Fig. 2C), the zinc and oxygen is clearly visible as the dark and bright spots, respectively. It is strongly related with their atomic masses and Z^2^ dependence of the signals^55^. Then, from the Fig. 2C it can be observed that zinc creates a nanoparticles core of the bio-ZnO NCs samples coated with the natural origin (bacterial metabolites) surface deposit.

The 1C and 4a_ZnO NCs and their corresponding elemental mapping associated with oxygen and zinc are presented in Fig. 2B. Both analyzed elements are homogeneously distributed on the surface through tested samples. It is highly related with the composition of nanoparticles core and surface deposit investigated by EDX detector (shown in the Fig. 2D). The major elements of 1C and 4a_ZnO NCs are oxygen (0.5 keV signal) and zinc (the 1, 8.5 and 9.5 keV signals). Additionally, some signals from phosphorus, chloride and copper were detected—the Cu might be associated with the grid composition used for the analysis (Fig. 2C). The SAED pattern showed next to the TEM micrographs of bio-ZnO NCs (Fig. 2D) confirmed the highly crystalline nature of the biologically synthesized nanoparticles, which is in a good correspondence with the data obtained during XRD analysis (Fig. 1).

**Figure 3:**
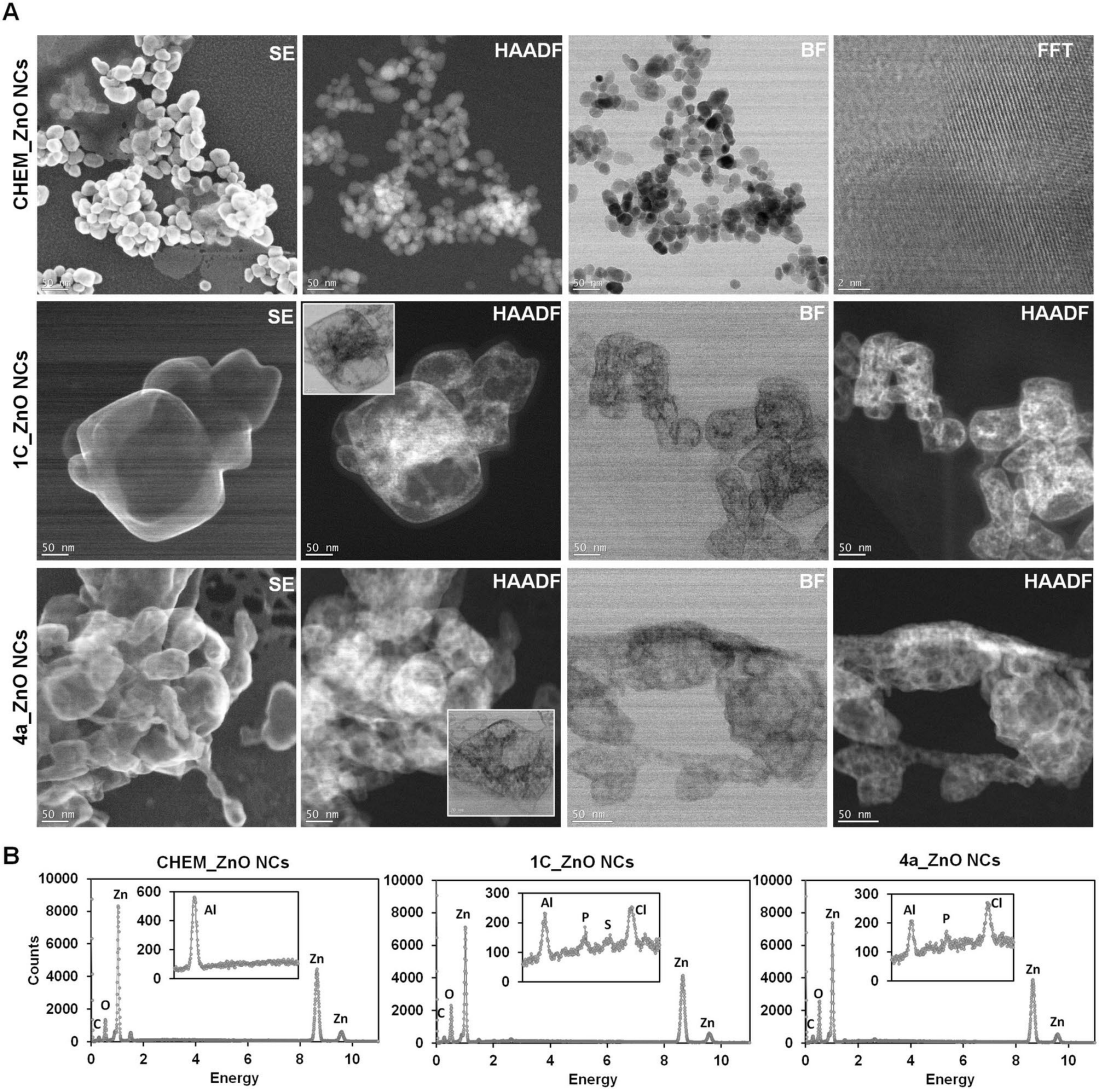
STEM images of all tested ZnO NCs recorded with SE, HAADF, BF and FFT detectors (**A**); the EDX spectra for CHEM, 1C and 4a_ZnO NCs (**B**).

For better understanding the core–shell structure of tested nanoparticles, the STEM microscopy with SE, HAADF, BF and FFT detectors were applied for CHEM, 1C and 4a_ZnO NCs (Fig. 3). In general, the STEM micrographs shows that all tested nanoparticles are homogenously dispersed into the organic matrix.

The SE-STEM micrographs (Fig. 3) confirmed the globular morphology of all tested ZnO NCs. Secondary electron imaging might be also helpful tool providing information about the coverage of nanomaterials by some adsorbates^56^. In the case of 1C and 4a_ZnO NCs, the nanoparticles with core and specific shell were observed. In addition, for 4a_ZnO NCs some agglomerates were also found.

The high HAADF detector provides qualitative information about the chemical composition of the samples. The contrast in the HAADF image is produced as a function of the atomic number of the elements in the sample. The technique is alternatively called Z-contrast. Heavy elements create high contrast on the HAADF detector (white zones), while light elements create little contrast (dark zones)^54,55^. In our study, the HAADF detector provides the improved imaging of bio-ZnO NCs core–shell structure. As visible from the Fig. 3, the Zn (*Z* = 30) atomic column is visible as a bright spots and indicates the zinc core of nanoparticles. At the same time, the O atomic columns (*Z* = 8) are practically invisible, because of the Z^2^ dependence of the signal^54,55^. The amount of detected zinc is comparable between CHEM and 1C, 4a_ZnO NCs. Nevertheless, the different distribution of this element could be noticed. In the case of CHEM_ZnO NCs, the organic coating around the nanoparticles core is visible. On the other hand, in 1C and 4a_ZnO NCs the coating it is take place in deferment mode; the zinc is homogenously distributed through whole organic matrix. The complementary information were provided by use of the bright field (BF) detector which allow for atomic detection of light and heavy elements)^57,58^. In this type of analysis, the opposite visualization is possible—Fig. 3 (BF mode) show a broad, dark spots corresponding to the same zinc atomic numbers (*Z* = 30) as in the HAADF images.

The appearance of organic deposit on the bio-ZnO NCs surface were also confirmed by EDX spectra showing the organic residues such as carbon, phosphorus and sulfur for 1C_ZnO NCs and carbon, phosphorus for 4a_ZnO NCs (Fig. 3B). In the case of CHEM_ZnO NCs, the zinc and oxygen were dominant detected elements (Fig. 3B). Additionally, STEM observations using the fast Fourier transform (FFT) detector enabled the visualization of layered arrangements of zinc atoms analogous to the crystallographic planes in the zinc oxide wurtzite structure, which is in an accordance to XRD data (Fig. 1A).

### Atomic force microscopy (AFM)

**Figure 4:**
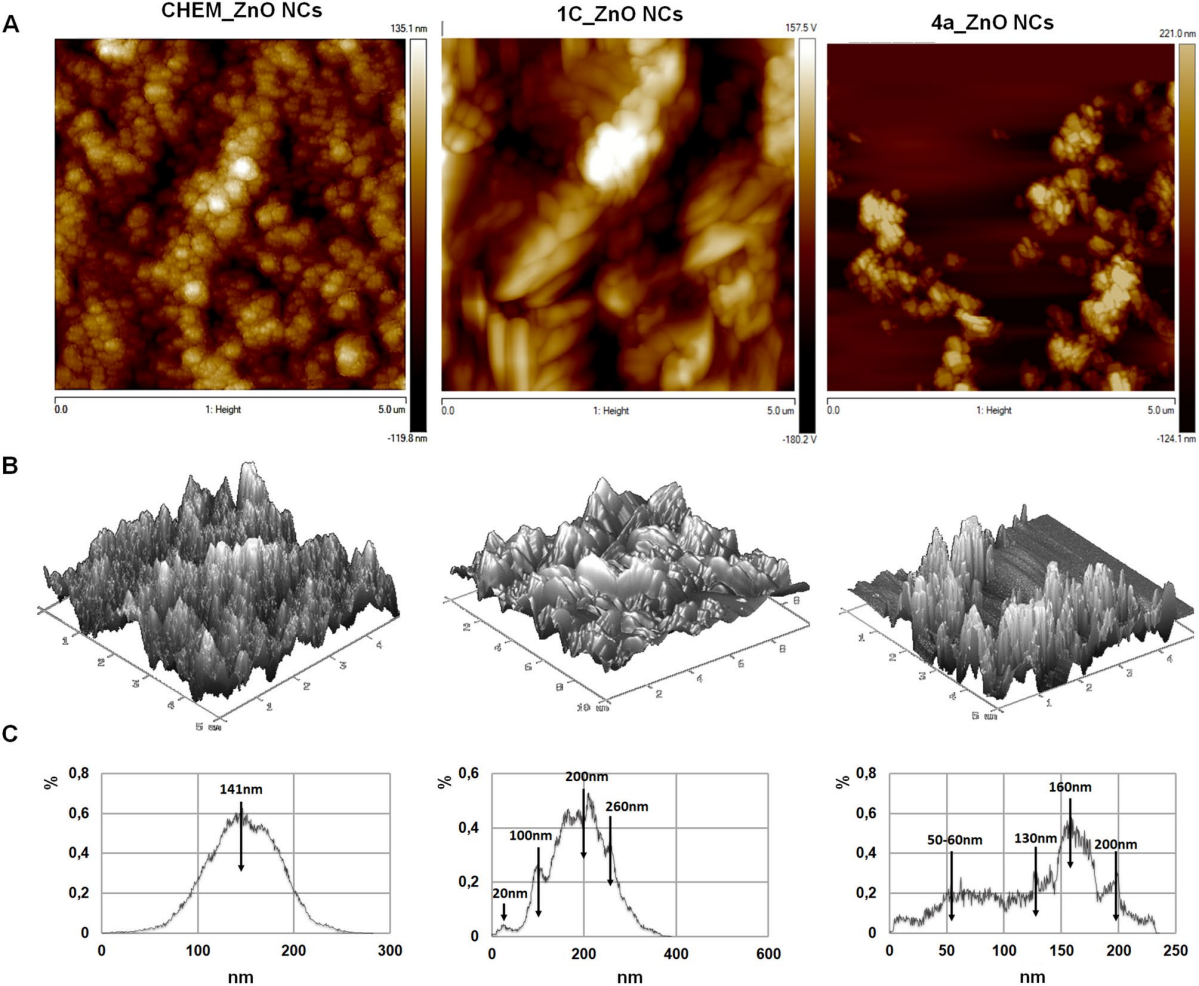
The AFM 2D (**A**) and 3D (**B**) images of chemically synthesized CHEM and biologically synthesized: 1C and 4a_ZnO NCs; the size population distribution (**C**).

The surface morphology of the all tested ZnO formulations were examined by atomic force microscopy (AFM) which is based on atomic level interaction between the tip and the samples. On the Fig. 4, the two-dimensional (2D) and three-dimensional (3D) AFM images with frame size of 5 µm obtained for the CHEM_ZnONCs, 1C and 4a_ZnO NCs are presented, respectively. In the 2D view, the intensity of the color reflects the height of the nanocomposites.

The AFM analysis of ZnO nanocomposites indicated that the type of synthesis method influenced the topography and size of nanocomposites. CHEM_ZnO NCs were characterized by homogenous surface topography and mono dispersed single size population measured as a 141 nm. On the other hand, bio-ZnO NCs (1C and 4A) were found to have rather a heterogeneous surface morphology with a few size population. For the 1C_ZnO NCs the dominant population is 200 nm size, whereas for the 4a_ZnO NCs it is 160 nm (Fig. 4C). Furthermore, the 2D AFM image of CHEM and 4a_ZnO NCs indicated that the nanocomposites approximately have a spherical shape (Fig. 4A). Contrary to them, the 1C_ZnO NCs might be described as an unequal rocky grains with higher amount of large pits and asperities. In the consequence, 1C_ZnO NCs were found to have a surface with more roughness, comparing to other samples. The increment of the ZnO NCs surface roughness is associated with the presence of different functional groups (organic coating) on their surface. In the case of the CHEM_ZnO NCs dispersed in butyl acetate, the surface roughness is lower than in the bio-ZnO NCs (1C; 4A) with the organic surface deposit coming from the bacterial metabolites that naturally were secreted during the inoculation step. Yang et al. have observed similar effect after the coating of titanium oxide nanoparticles (TiO_2_ NCs) with the bovine serum albumin (BSA)—it resulted with the higher roughness of tested surfaces. The surface roughness seem to be a crucial physicochemical parameter which might influence the therapeutic efficacy of NCs- based formulations by affection on the cell uptake and intracellular interaction of used nanoparticles^59^. Xue with colleagues^60^ have prepared novel core‒shell hybrid silica nanoparticles and manipulate their surface roughness by coating with polydopamine. They have found out that the increased surface roughness of obtained NCs significantly raised the cellular internalization, and in the consequence, the inhibition of tumor cells growth was observed. Then, it can be assumed that 1C and 4a_NCs biologically synthesized nanocomposites in this study and characterized by higher surface roughness than CHEM_ZnO NCs have the potential to be promising medical agent.

For all types of tested nanomaterials, the line and grain analysis reveals that the nanocomposites are in the range of nanometer, which is consistent with the electron microscopic data. The 2D and 3D views of the AFM pictures suggest also that CHEM_ZnO NCs were well dispersed with no apparent aggregation, while the bio-ZnO NCs consist of both, a uniform deposited layer and some amount of aggregates (Fig. 4A, B). The explanation of observed effect might be fact that the dispersion of the chemically synthesized ZnO NCs, then, dispersed in butyl acetate are coated one by one in the same way. While, the 1C and 4a_ZnO NCs aggregation could be attributed to the presence of natural origin coating of their surface. Moreover, different density of the particles is correlated to the nanocomposites size: CHEM_ZnO NCs around 140 nm (spherical shape), 1C_ZnO NCs—around 200 nm (unequal rocky grains) and 4a_ZnO NCs—predominant 160 nm (spherical shape). According to the fact that nanocomposites in their structure contained organic deposits will have a tendency to aggregate and generate different roughness surface.

To sum up, complementary information about the size distribution of the chemical and biological ZnO NCs were determined by different approaches from the X-ray diffration (crystallite size), electron and atomic force microcopy (grain size) to dynamic light scaterring (DLS; hydordynamic diameter). Then, the differences between the ZnO NCs’ size estimated from the different techniques can be attributed to the fact that the particles are formed from more than one crystallite and sue to existence of core–shell NCs structure. The results from XRD proved the wurtzite structure of all tested ZnO NCs (Fig. 1A). Electron microscopy (SEM, TEM, STEM) with different detectors confirmed the nanoparticle core surrounded by organic coating. Moreover, AFM analysis supplement this view with the size distribution and AFM 2D and 3D imaging showing both the vertical and lateral distribution of height. The equivalent diameters of a grain of nanocomposites was calculated from the full width at half maximum (FWHM) and comparable with the average size of nanoparticles recorded by TEM analysis where have been noticed the presence of twin and multiple twinned particles in form of crystallite. Notable to specify the fact that was applied two different techniques to prove that synthesized nanocomposites consist of nanoparticles and organic core. Additionally, this fact has been proven by another complimentary techniques such as SEM/STEM. Finally, as far as the bio-ZnO NCs with surface deposit might be characterized as biocolloids, the DLS approach was applied to measure their hydrodynamic diameter (discussed later).

### Organic surface deposit (FTIR-ATR, LDI-MS)

Fourier transform infrared (FTIR-ATR) spectroscopy was performed to identify and classify probable biomolecules that can be reliable for coating the bio-ZnO NCs, leading to presence of specific organic deposit on their surface. However, this technique has some limitation as far as it is possible just to predict the dominant active groups. Then, in order to extend the applicability of FT-IR spectroscopy to deposit characterization, we also present a more accurate mass spectrometry analysis including LDI-TOF-MS (described later).

The Fig. 1B illustrates the FTIR-ATR spectra registered in the ν = 400–4000 cm^−1^ range. Spectroscopic analysis were carried out on all ZnO NCs samples (both chemically and biologically synthetized). As seen in the Fig. 1B, the zinc oxide were identified in all of them, as the Zn−O bond (525 cm^−1^; band no. 7). The differences between tested nanoparticles occurred in the infrared region of 1350–1800 cm^−1^ which is typical for the amide I (1600–1800 cm^–1^), II (1470–1570 cm^–1^) and III (1250–1350 cm^–1^) sidechain vibrations^61–63^.

The CHEM_ZnO NCs spectrum exhibited several peaks at 1566 (1a), 1411 (2), 1304 (4) and 1098 (5a) cm^−1^. As far as we know that CHEM_ZnO NCs were dispersed in butyl acetate (according to the manufacturer information), the registered peaks are characteristic for some organic residues and polymers. Accordingly, the peak at 1566 cm^−1^ (1a) is characteristic for the stretching vibration of the carbonyl group in an acetate molecule^64^. The broad stretching band at 1411 cm^−1^ (2) might be a result of the methyl group (–CH_3_) vibrations which confirms the presence of intact acetate ligands (CH_3_COO^–^)^65,66^. Another peak (1304 cm^−1^) might derived from the C–C–O stretch vibration of butyl acetate used for the NCs stabilization^67^, while the presence of vibrations at 1098 cm^−1^ (5a) demonstrates the stretching vibrations of the C–O groups^68^. In comparison to biologically obtained nanoparticles, the lack of the band no. 3 were observed. Furthermore, the CHEM_ZnO NCs did not show the band around 800–850 cm^−1^ (6) which was detected for the 1C and 4a_ZnO NCs.

In the case of biologically synthetized ZnO NCs (1C and 4a_ZnO NCs), the increase of the signal intensity over the entire spectral range was observed. The observed signal at 1638 cm^−1^ (1) correspond with the *ν*(C = O) or δ(NH_2_) vibration of glutamine/asparagine (Gln/Asn)^63^. Moreover, the band position around 1630–1640 cm^−1^ is specific for the *ε*-NH_2_ group of lysine (Lys)^69^. The peak localized at υ = 1375 cm^−1^ (4) is characteristic for the *δ*(CH) and *ν*(CC) vibrations of serine, but might be also assigned to the *δ*(CH_3_) of valine^70^. In the range around 1100–1200 cm^−1^ (5a), the C–C stretching and N–H bending vibrations occurred^70^. Additionally, this region is also characteristic for metabolites produced by lactic acid bacteria^71^. The band no. (6) occurring around 820 cm^−1^ might be assigned to the imidazole ring of histidine (His) as well as to the anomeric region of carbohydrates^72^. The low intensity of FTIR-ATR spectra registered for the CHEM_ZnO NCs as well as the lower number of detected peaks might be a reason of butyl acetate ZnO NCs dispersion. On the other hand, biologically synthetized nanoparticles had the specific organic deposit on their surface mainly originating from the bacterial metabolites and proteins. The presence of natural origin bio-NCs coating is in a good agreement with our previous study^13^ also describing the extracellular ZnO NCs synthesis using *L. paracasei*.

For the better understanding of the ZnO NCs surface coating composition, the laser desorption/ionization with time of flight analyzer mass spectrometry (LDI-TOF-MS) was applied. Our previous experiences showed that the use of LDI-TOF-MS is an effective tool for description of bio-NCs organic deposit composition^13,27^.

**Figure 5:**
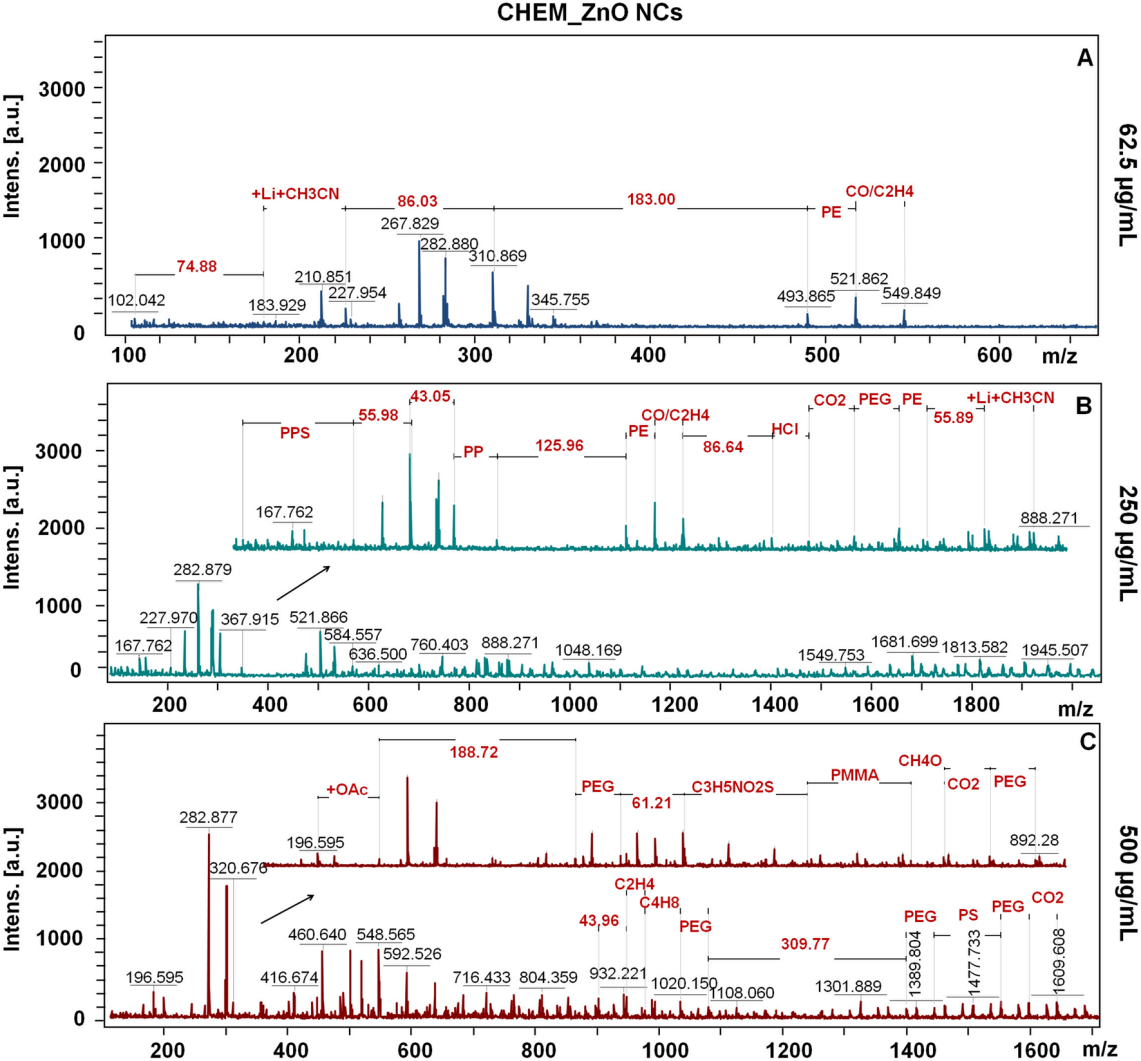
The LDI-TOF-MS spectra for core–shell signals of CHEM_ZnO NCs at 62.6 (**A**), 250 (**B**) and 500 (**C**) µg/mL concentration.

**Figure 6:**
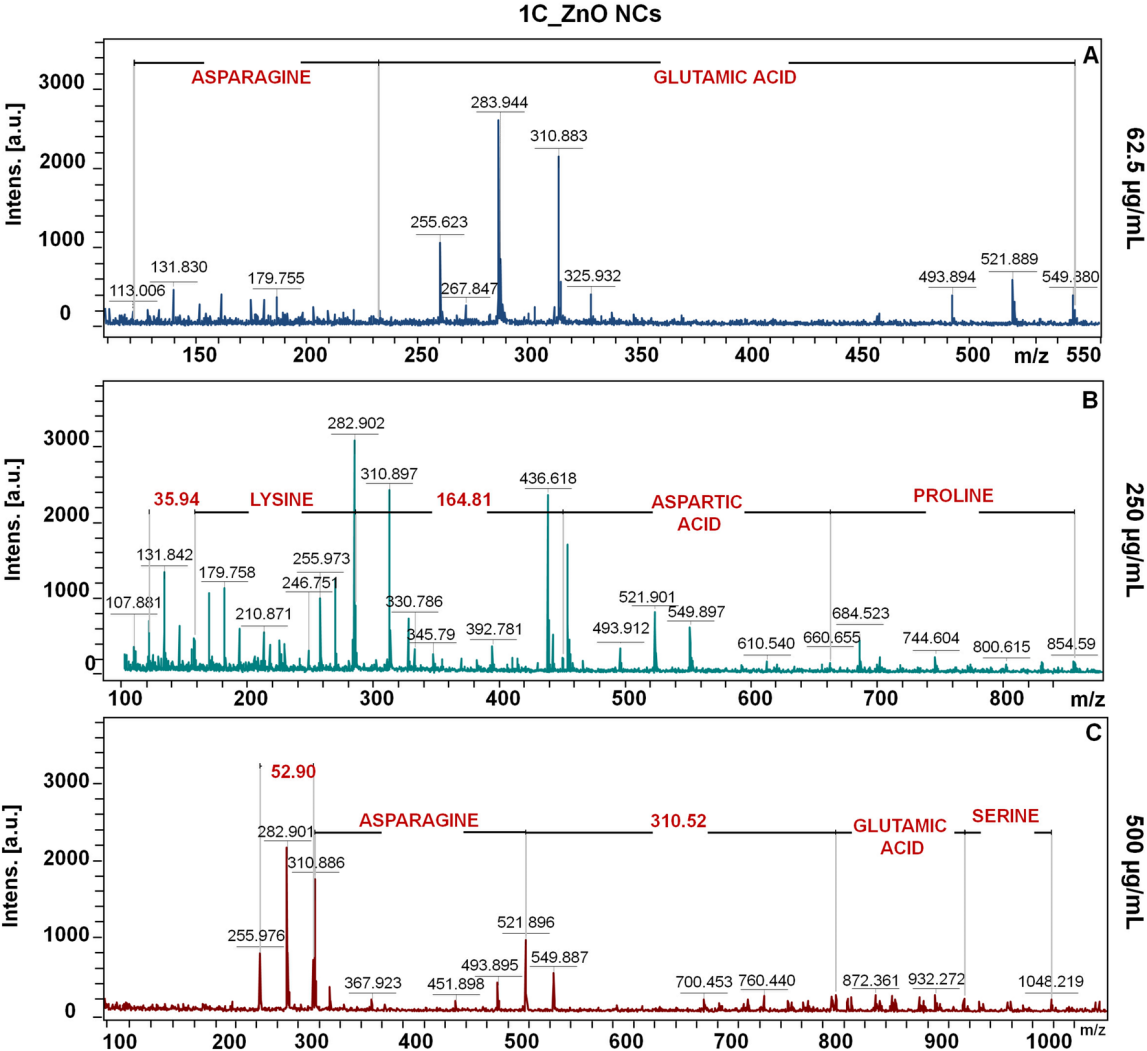
The LDI-TOF-MS spectra for core–shell signals of 1C_ZnO NCs at 62.6 (**A**), 250 (**B**) and 500 (**C**) µg/mL concentration.

**Figure 7:**
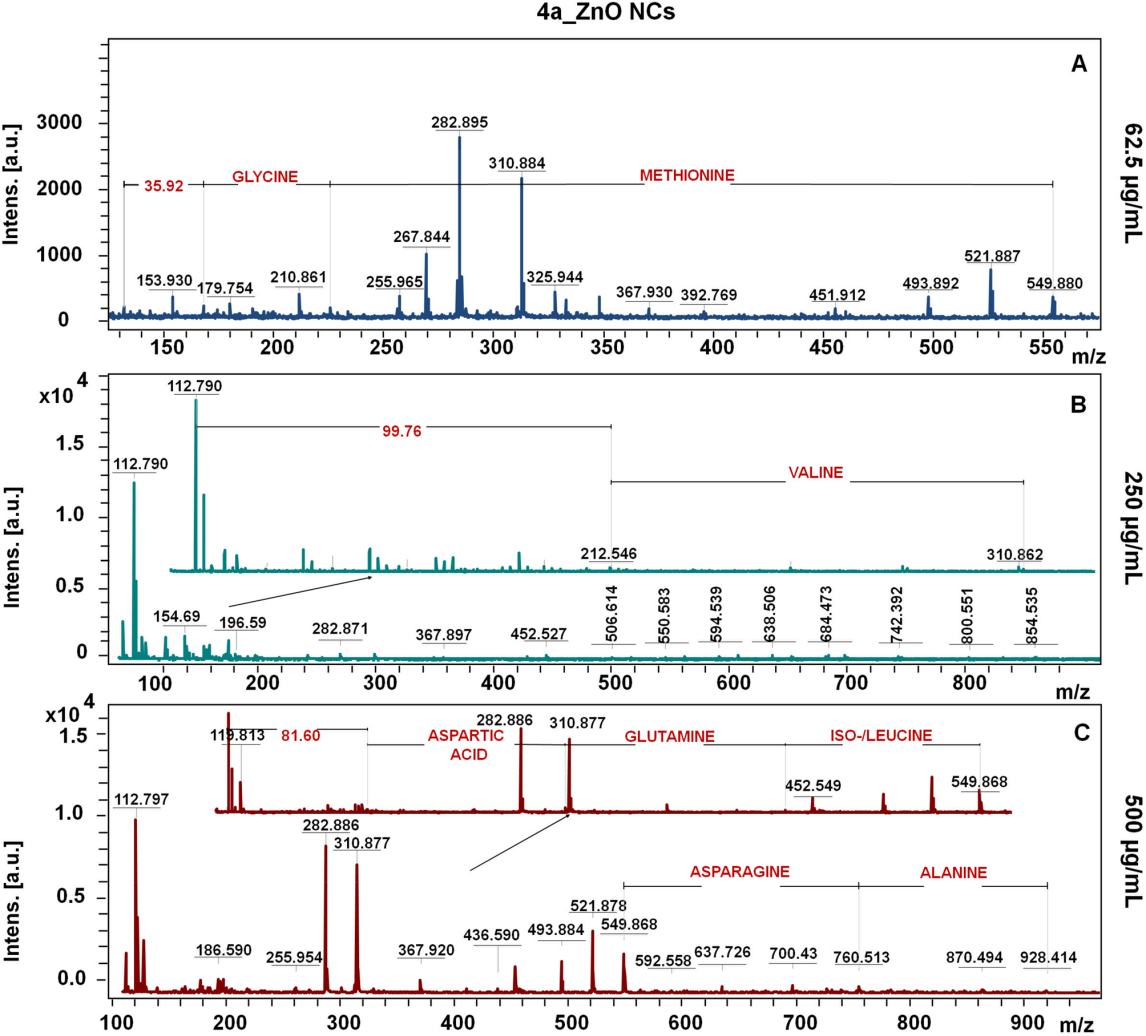
The LDI-TOF-MS spectra for core–shell signals of 4a_ZnO NCs at 62.6 (**A**), 250 (**B**) and 500 (**C**) µg/mL concentration.

The Figs. 5, 6 and 7 presents the molecular fingerprint of chemical and biological ZnO nanoparticles. The LDI-TOF-MS analysis was carried out in dependence on ZnO NCs concentration (62.5, 250 and 500 µg/mL). With the increasing nanoparticles concentration, the number of detected signals and their intensity were considerably higher. Besides the larger quantity of NCs organic surface coating (which was also observed by photoluminescence spectra), this effect might be correlated with the semi-conductive properties of zinc oxide. ZnO NCs by absorbing energy from the LDI laser are able to accomplish effective desorption and ionization as a novel type of matrix.

For the CHEM_ZnO NCs at the lowest concentration (62.5 µg/mL), some organic residues such as carbon monoxide/ethen (CO/C_2_H_4_) or polymers (PE) were detected (Fig. 5). All of detected signals might derive from the organic dispersant provided by the CHEM_ZnONCs NCs manufacturer. According to the literature and MS libraries, the most common butyl acetate (BuAcO) MS signals are detected for the *m*/*z* = 43, 56, 61 and 74, respectively ^73^.

Then, the signals on the LDI-TOF-MS spectra (Fig. 5A) at *m*/*z* = 74.88 and 86.03 might be correlated with the one and two (2 × *m*/*z* = 43) fragments of butyl acetate, respectively. Additionally, the signals at 183 were observed. Since, the most abundant zinc isotope is ^64^Zn, those signals might correspond to fragment of butyl acetate and two zinc molecules. With the increasing CHEM_ZnO NCs concentration (Fig. 5B and C), the number of detected polymers (i.e. PP, PE, PEG, PPS, PMMA) and organic residues (i.e. CO_2_, C_4_H_8_, CH_4_O) also increased. Taking into consideration manufacturer information, chemical ZnO NCs are not functionalized or stabilized. However, from the detailed spectroscopic and spectrometric analysis showed that they have some polymers on their surface. What is more, some butyl acetate fragments (*m*/*z* = 43.05, 55.98 and 86.64) as well as zinc molecules attached to butyl acetate (*m*/*z* = 125) were registered (Fig. 5B). The same trend was maintained for the highest concentration (Fig. 5C), but even more zinc molecules linked to butyl acetate were observed (*m*/*z* = 188.72 for 2 zinc and one BuAcO fragment and *m*/*z* = 309.77 for 3 zincs and two BuAcO fragments).

Speaking of biologically synthetized nanoparticles—for both, 1C and 4a_ZnO NCs, the amino acid residues were the major part of surface deposit (Figs. 6 and 7), what is in a good correlation with the FT-IR and PL spectra, as well our previous work^13^. For the 1C_ZnO NCs at the lowest concentration (Fig. 6A), only two amino acids (asparagine and glutamic acid) were detected. For the 250 and 500 µg/mL, the number of detected signals assigned to amino acids increased. Interestingly, at 250 µg/mL concentration, between the lysine and aspartic acid, the two zinc molecules with water was detected (*m*/*z* = 164.81). Also, the lysine residue was linked with two molecules of H_2_O (m/z = 35.94) (Fig. 6B). Next, the organic deposit of 1C_ZnO NCs at highest concentration (Fig. 6C) were found to be composed by such amino acids as asparagine, glutamic acid and serine. Moreover, the signals registered at m/z = 52.90 and 310.52 indicates the presence of three water molecules as well as the four Zn linked to three H_2_O, respectivelyFor the 4a_ZnO NCs at 62.5 µg/mL (Fig. 7A), the methionine and glycine are linked with two molecules of water (*m*/*z* = 35.92). With the increase of concentration, beside the H_2_O, some zinc was detected. Similarly, for the concentration of 250 µg/mL (Fig. 7B) signal at *m*/*z* = 99.75 was assigned to the one zinc linked with two water molecules. In turn, the molecular fingerprint for the 4a_ZnO NCs at 500 µg/mL (Fig. 7C) is characterized by the following peptides: aspartic acid–glutamine–leucine connected with one Zn and one H_2_O (*m*/*z* = 549.86) and asparagine–alanine (*m*/*z* = 928.41). The slight differences between two types of bio-ZnO NCs are only due to the type of amino acids detected during mass spectrometry analysis. Some of them, like asparagine or aspartic acid are common for 1C and 4a_ZnO NCs. In turn, another occurs only for 1C_ZnO NCs (such as proline) and for 4a_ZnO (such as valine or leucine). However, all of differentiating residues belong to the same, non-polar, amino acid group. Then, it can be assumed that two different probiotic strains used for the synthesis produce similar metabolites allowing for coating the ZnO NCs. Also, Similar bio-ZnO NCs molecular fingerprint were detected in our previous work^13^, where another probiotic strain (*Lactobacillus paracasei* LC20) were used for the extracellular synthesis. The organic surface deposit consisted mostly of amino acids such as glycine, asparagine, glutamic/aspartic acid and leucine. What is important, also in this case, the zinc and H_2_O molecules were observed, which point out the comparable mechanism of bio-ZnO nanoparticles formation and coating^10,13^.

### Optical properties of ZnO NCs

**Figure 8:**
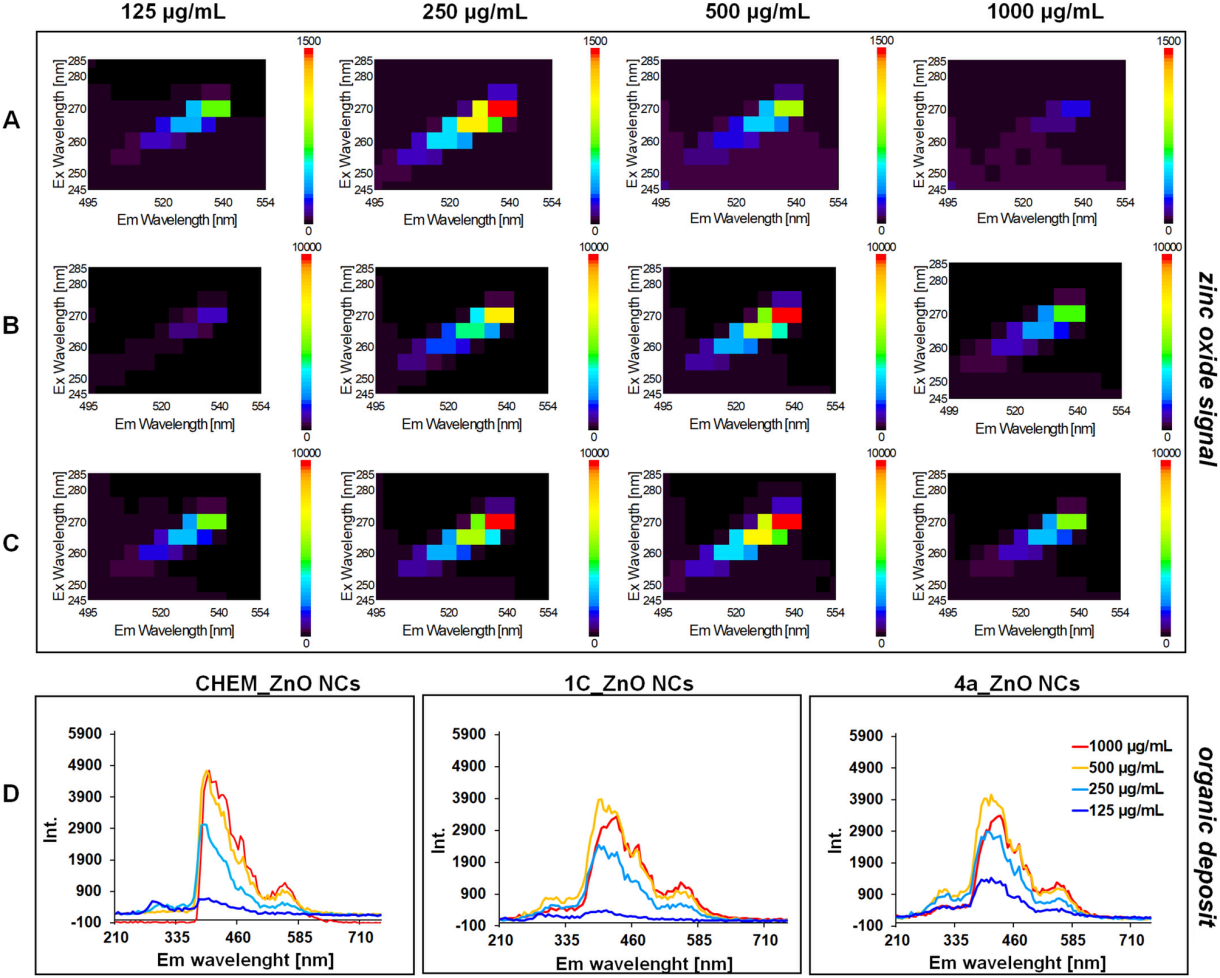
The photoluminescence spectra at two specific excitations regions (λ = 540 nm and λ = 420 nm) for CHEM_ZnO NCs (**A**), 1C_ZnO NCs (**B**) and 4a_ZnO NCs (**C**) at different concentration; organic deposit of tested NCs (**D**).

In order to study the optical properties and detection of the organic surface deposit in the all ZnO NCs samples, photoluminescence (PL) spectra were recorded in the 210–735 nm range, for the different nanoparticles concentration (125, 250, 500 and 1000 µg/mL). Zinc oxide has two emission bands: (1) a narrow exciton emission band at a wavelength about 380 nm corresponding to the near band gap and (2) a wide, intense band at a wavelength about 535 nm, which is assigned to the ZnO trap state^74,75^. The Fig. 8 presents the PL spectra for chemical and biological ZnO NCs. In all samples, it was observed that the excitation of nanoparticles with a wavelength of λ = 270 nm resulted in the appearance of a distinct emission peak around λ = 540 nm; this is a green emission peak characteristic for the singly ionized oxygen vacancy (*V*_*O*_) in zinc oxide^75–77^. Another important signal was registered at emission λ = 420 nm which might be related with the presence of organic coating on the ZnO NP surface. Such amino acids as tryptophan (Trp), tyrosine (Tyr) or phenylalanine (Phe) are often described as natural fluorescent residues, due to their specific fluorophores undergoing the π–π transition under light excitation^78,79^. It has also been reported that some peptides are able to display a photoluminescence with the main emission peaks at λ ≈ 400 and 450 nm^80–82^.

The intensity of those two described the PL signals varied in dependence on the ZnO NCs concentration. The CHEM_ZnONCs exhibited the highest zinc oxide peak (270; 540 nm) intensity at 250 µg/mL concentration while 1C_ZnONCs and 4a_ZnONCs at 500 µg/mL. The increase of the green light emission intensity might be correlated with the size and stability of nanoparticles. In the smaller and more stable ZnO NCs, the greater fraction of oxygen vacancies is presented, and in the consequence, the intensity of emission peak λ = 540 nm is increased. In the case of the highest NCs concentration (1000 µg/mL), the suppression of zinc oxide signal was observed. Beside the colloidal stability of nanoparticles, which is strongly related with their concentration, the observed phenomenon might be also associated with the quantity of the organic residues on the ZnO NCs surface.

Sharma et al. have observed that the green luminescence of chemical ZnO nanoparticles is originating from certain groups presented on their surface (mostly hydroxyl and acetate groups) coming from the sol–gel synthesis method. In our study, we have analyzed the chemical NCs dispersed in the butyl acetate and the bio-ZnO NCs obtained through extracellular synthesis with natural origin organic surface groups.

As shown in the Fig. 8, the intensity of photoluminescence peak assigned to organic deposit increased proportionally to the ZnO NCs concentration. Chemically obtained nanoparticles exhibited the most intense spectra at 500 and 1000 µg/mL. On the other hand, the intensity of λ_em_ = 420 nm was the highest on the 500 µg/mL for both, 1C and 4a_ZnO NCs. Then, it can be concluded that with the higher concentration, the amount of surface organic residues also increase considerably. For the CHEM_ZnO NCs, the λ_em_ = 420 nm signal might be related to the organic residues of butyl acetate coating. Carolan and Doyle^83^ have observed the broad photoluminescence signal at emission λ = 420 nm for germanium nanocrystals coated with the acetate groups. Nanostructures are characterized high surface to volume ratio which might resulted in a change in the coordination environment of the surface atoms/ions in relation to those inside the nanoparticles^84^. Due to numerous point defects (electron and atomic) and surface defects (e.g. dislocations), there is a significant amount of unsaturated coordination sites on the surface of such systems, which results in a change in the spectroscopic properties of such nanomaterials^85^. What is interesting, according to the literature, an increase in the number of ZnO NCs surface defect trap states might degrade the photoluminescence^86–88^. Considering the NCs surface coating, some functional groups presented on the nanoparticles deposit may have different energy levels and produce a specific amount of emission trap states. Lou et el.^89^ have explained that the detachment of binding ligands would lead to the exposure and increase of surface defect trap states. In the consequence, the degradation occurred due to the capture of excitons by these defects^89^. For this reason, the increment of organic surface deposit signal (λ_em_ = 420 nm) might be related with the lower photoluminescence characteristic for the zinc oxide signal (λ_em_ = 540 nm). The broadening of the fluorescence emission band is a direct result of the higher size of the ZnO NCs system in the solvated water system and the influence of the organic coats. Moreover, the signals registered in the 210–350 nm range are less intensive however still are detected. The fluorescence generated in the respective range correspond to emission by organics coats of the aromatic side-chains (Tryptophan, Tyrosine, Phenylalanine) residues of peptides at ~ 300 nm.

The interaction between tested ZnO NCs and organic groups presented on their surface might enhance the ability for efficient charge carrying for the photo-generated electron hole (e^−^/h^+^) pairs^90^. In the consequence, a photocatalytic activity enhancement under exposed to sunlight is observed.

The photocatalytic activity of chemical and biological ZnO NCs was evaluated against the methylene blue (MB) dye as the degradation efficiency (E) calculated using the following equation:

$$E = \frac{{(A_{0} - A_{1} )}}{{A_{0} }} \times 100\%$$

where A_0_ is the absorbance of the MB without the ZnO NCs (control), A_1_ is the absorbance of MB dye solution with the ZnO NCs after sunlight irradiation in time.

**Figure 9:**
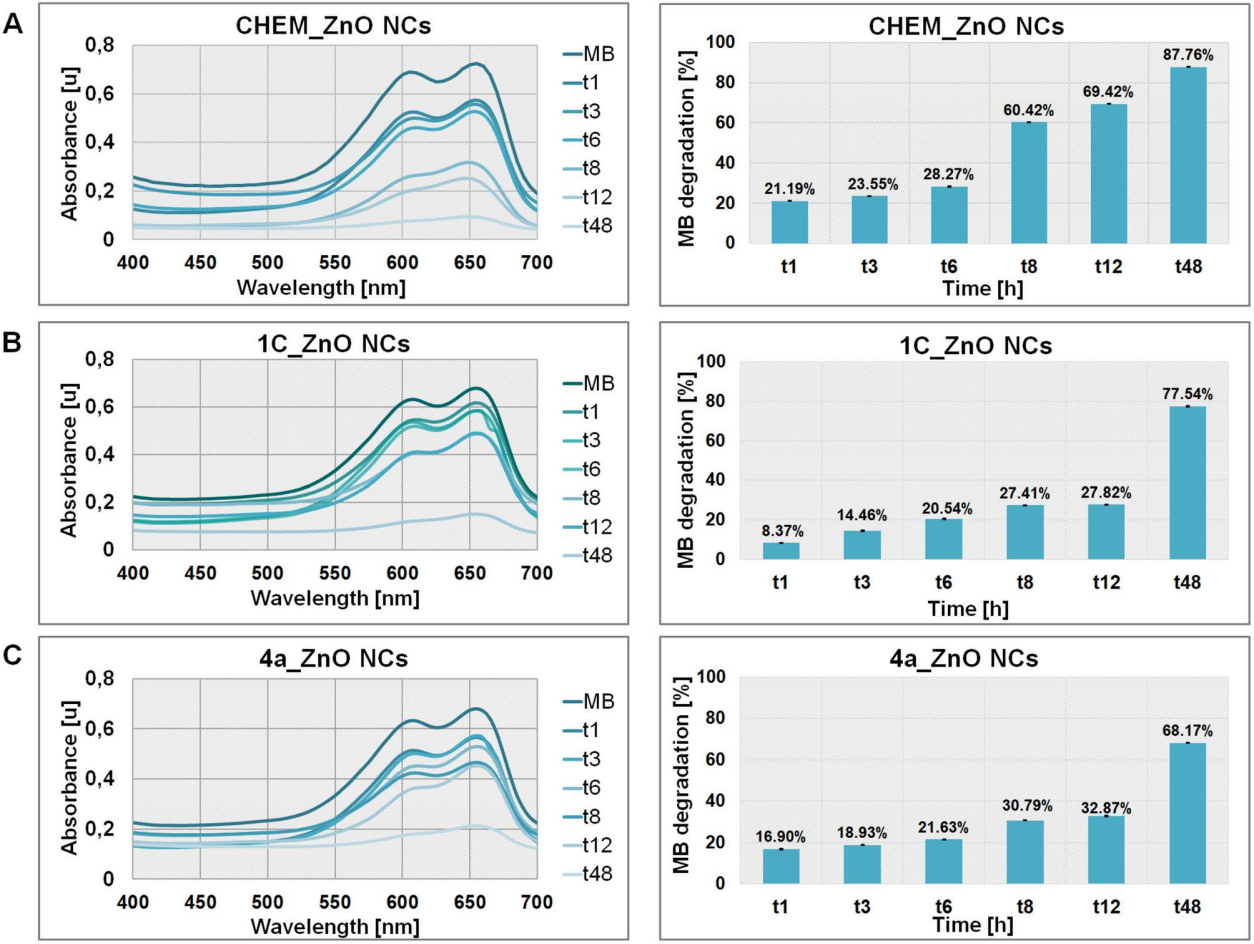
The UV–vis plot and efficiency of MB photocatalytic degradation (%) after CHEM_ZnO NCs (**A**), 1C_ZnO NCs (**B**) and 4a_ZnO NCs (**C**) treatment.

The change in the MB absorption intensity during the time intervals (1, 3, 6, 8, 12 and 48 h) and after the ZnO NCs treatment is presented in the Fig. 9. The characteristic peak for methylene blue was registered at 665 nm and is related with the hetero-polyromantic linkage of this dye. Experimental data shown in the Fig. 9 indicated that the major MB peak (665 nm) intensity decreased during the sunlight irradiation, which confirm the dye photodegradation. In the case of CHEM_ZnO NCs, around 70% of MB was degraded after 12 h of photocatalysis. On the other hand, the treatment of MB with both, 1C and 4a_ZnO NCs, resulted in the photodegradation at 28 and 33% level, respectively. Finally, after the 48 h of the process, the efficiency of methylene blue photodegradation reached around 88, 76 and 68% for using accordingly CHEM, 1C and 4a_ZnO NCs. Then, the chemical nanoparticles might be considered as a better and more efficient photocatalyst, compared to the biologically synthetized ZnO NCs. Comparable data were collected in our previous work^29^ where the chemical ZnO NCs exhibited stronger photocatalytic activity toward MB dye at sunlight and UV irradiation. The differences between nanoparticles photocatalytic action might be strongly associated with their size, stability and their surface organic deposition. Some research works have presented the strong dependence of photocatalytic activity and increasing size of NCs^91–93^.

Another papers^94,95^ underlined the strong enhancement of ZnO photocatalytic properties after surface modification. In contrary, Leung et al.^96^ have proved that surface modification of ZnO NCs resulted in the lower photodegradation of methylene orange. Therefore, it is important to consider this factor regarding the further application of nanomaterials. In the case of our study, the natural origin coating of 1C and 4a_ZnO NCs allow for the MB degradation, but the uncoated chemical ZnO NCs exhibited stronger photocatalytic properties.

The mechanism of ZnO NCs photodegradation activity might be explained by their electron structure. Zinc oxide have an electron-filled valence band (VB), and an unfilled conduction band (CB). The energy difference (ΔE) between these bands, defined as the band gap, is the amount of energy that needs to be supplied for an electron to move from one band (VB) to another (CB)^90,97^. For zinc oxide, the band gap is 3.36 eV^90^, and this energy is provided by photons of electromagnetic radiation with a wavelength λ < 400 nm. The radiation causes the electron (e^–^) to go to the conduction band, leaving a positively charged so-called electron hole (h^+^) and thus creates a hole-electron pair (h^+^–e^–^). This state, called the exciton, is unstable and exhibits strong redox properties. The positively charged electron holes and excited electrons react with e.g. water molecules or hydroxyl ions leading to the formation of reactive oxygen species (ROS), such as hydroxyl radicals (OH^•^), superoxide anion radicals (O_2_^- •^) or hydrogen peroxide (H_2_O_2_)^97,98^. These formed electron–hole pair may permit the direct oxidation of methylene blue or another dye to photodegraded intermediates.

### Size and Stability of ZnO NCs

The presence of organic deposit on the tested ZnO NCs surface confirmed by spectroscopic and spectrometric methods allow to described both, chemical and biological, nanoparticles as a biocolloids with a surface charge. Then, the zeta potential (ζ; ZP) and hydrodynamic diameter measurements were applied to investigate the colloidal stability of CHEM, 1C and 4a_ZnO NCs, respectively. All analysis were performed depending on the NCs concentration (62.5, 125, 250, 500 and 1000 µg/mL) and monitoring time (after 1, 2, 4 and 7 days).

**Figure 10:**
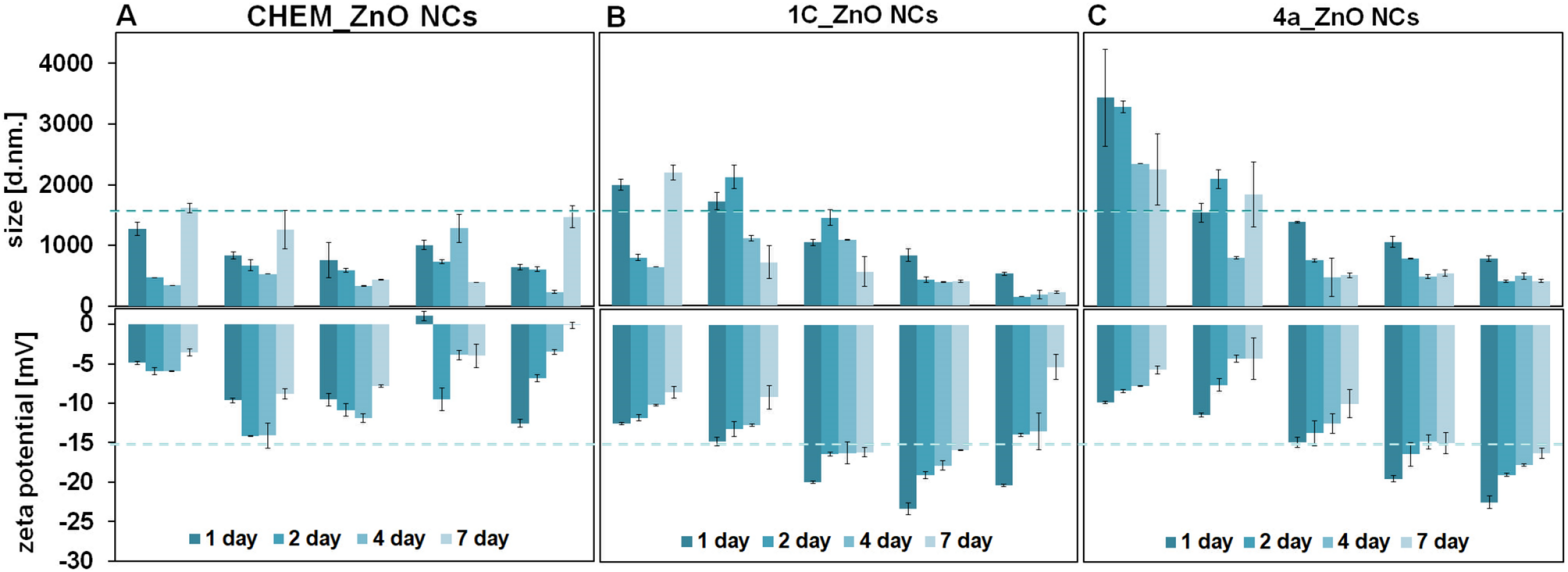
Study of the CHEM_ZnO NCs (**A**), 1C_ZnO NCs (**B**) and 4a_ZnO NCs (**C**) shown for hydrodynamic size (top) and zeta potential (bottom) for 7 days.

As presented in the Fig. 10A, CHEM_ZnO NCs were found to have hydrodynamic diameter from 233 nm (at 62.5 µg/mL concentration) to 1620 nm (at 1000 µg/mL concentration). In the contrast, the biologically synthetized ZnO NCs were found to have hydrodynamic diameter from 184 to 2200 nm and from 405 to 3400 nm for 1C and 4a_ZnO NCs, respectively (Fig. 10B and C). In general, the nanoparticles hydrodynamic diameter and tendency to create aggregates is strongly related with two tested factors—concentration and time as well as with the zeta potential value. At the highest concentration (1000 µg/mL), all ZnO NCs exhibited the lowest colloidal stability during all 7 days of monitoring. For all tested samples, the higher hydrodynamic size was associated with the lower zeta potential value. The Fig. 10A shows that the CHEM_ZnO NCs had a hydrodynamic size around 1620 nm and ZP at − 3.5 ± 0.4 mV after 7 days at 1000 µg/mL concentration level. In contrary, the concentration of 250 µg/mL were found to be the most stable as far as the hydrodynamic size of CHEM_ZnO NCs were the lowest (430 nm) and the ZP value occurred around − 10 mV during all 7 days (Fig. 10A). Compared to CHEM_ZnO NCs, extracellularly synthesized nanoparticles show higher stability—both over time and depending on the concentration of ZnO NCs. In both cases (4a and 1C_ZnO NCs), the highest stability was determined for nanoparticles with a concentration of 62.5 μg/mL on the 1st day of measurements. The ZP values were then − 12.5 ± 0.5 mV for CHEM_ZnO NCs and − 22.4 ± 0.8 mV as well as − 20.3 ± 0.2 mV for 4a and 1C_ZnO NCs, respectively (Fig. 10). With the passage of time (from the 1st to the 7th day of measurements), the stability of bio-ZnO NCs slightly decreases—for the concentration of 125 μg/mL, the ZP value changes from − 19.5 ± 0.4 mV (1 day) to − 14.9 ± 1.3 mV for 4a_ZnO NCs (Fig. 10C). Comparing two types of bio-nanoparticles, it can be noticed that the 4a_ZnO NCs were moderately more stable at higher concentration (250 µg/mL) for 7 days of monitoring (Fig. 10C). Additionally, the ZP results demonstrate that the magnitude of zeta potential of bio-ZnO NCs increase to higher values with the decrease in nanoparticles concentration during the investigation. However, no considerably differences between 1C and 4a_ZnO NCs were observed—the bacterial strain used for the biosynthesis had no significant impact on further nanoparticles colloidal stability. The negative surface charge of 1C and 4a_ZnO NCs might be associated with the binding affinity of bacterial supernatant compounds with the nanoparticles surface. Moreover, the determined size from the AFM analysis is correlated to the DLS size. Slightly differences are determined by the operated protocol of each technique. Both, AFM and DLS determined the size of nanocomposites not size of nanoparticles as in case of TEM; the AFM measured based on full width at half maximum whereas DLS in hydrodynamic radius of solvated ZnO NCs with organics coats. Moreover, the used of complimentary approach it is explained by the fact that the nanocomposites synthesized by the biological method were found to be a complex structure consisted from the nanoparticles core and organic surface that naturally coated the NCs core.

The biocolloids aggregation processes are described by the thermodynamic Derjaguin-Landau-Verwey-Overbeek (DLVO) theory which include the attractive van der Walls and repulsive electrostatic double layer (EDL) forces^99,100^. According to the classical DLVO theory, colloidal stability increases with increasing net surface charge^99–101^. For the zinc oxide nanoparticles, the surface charge strongly dependent on the solution pH. At pH values near the isoelectric point (neutral charge), the nanoparticles EDL repulsion forces are lower than attractive van der Walls forces leading to aggregation^101,102^. In our previous works^12,13^ we have determined the influence of pH on the bio-ZnO NCs zeta potential.

Besides the factors such as pH, ionic strength or NCs concentration, it is also well known that surface properties such as coating or stabilization have a significant impact on NCs hydrodynamic diameter and aggregation. Among the commonly applied chemical stabilizers we can distinguish two main types of their action—(1) electrostatic repulsion and (2) steric mechanism^103,104^. The first one involves surrounding nanoparticles with ions with a charge opposite to their surface charge. As a result, a double layer is created consisting of different charges that stabilize the nanoparticles^105^. The second mechanism of action, mostly occurs through the addition of a polymer which, on the basis of physical or chemical adsorption, adheres to the nanoparticle blocking its surface. In the consequence, the increase of osmotic repulsive forces is observed and the stability of nanoparticles is enhanced^106,107^. Li et al.^44^ have determined the PEG-400 concentration effect on the chemical ZnO NCs aggregation level. Their data have indicated that the 50% concentration of stabilization agent caused the high aggregation of nanoparticles. On the other hand, Cai and Yao^108^ have produced the gold nanoparticles (Au NCs) with five different amino acid (aspartate, glycine, leucine, lysine, and serine) surface coatings. The ZP and DLS analysis data revealed that the amino acids deposition effectively reduced the aggregation of the Au NCs in DMEM culture medium. In the same way, Feng with colleagues^109^ have used valine, aspartic acid and lysine to modify the selenium nanoparticles (Se NCs) surface. The modified with amino acids Se NCs exhibited better dispersion stability than unmodified ones. The work of Al-Kordy et al.^19^ confirmed that metabolites in *Alkalibacillus* sp.W7 supernatant acted as capping and stabilizing agents for bio-ZnO NCs (zeta potential at 27.5 mV value). However, to the best of our knowledge no studies have been published on the bio-ZnO NCs natural surface organic deposit and its influence on the NCs colloidal stability (compared to chemical one). In our work, nanoparticles synthetized via extracellular microbiological approach demonstrated higher the zeta potential absolute values than the commercially available chemical ZnO NCs. The zeta potential of 1C an 4a_ZnO NCs at 62.5 µg/mL concentration were found to be − 20.3 and − 22.45 mV, respectively which indicates the presence of negatively charged groups on their surface. This negative value states the electrostatic repulsion among the biosynthesized nanoparticles.

As shown by the spectroscopic and spectrometric data (Figs. 1B and 5, 6 and 7), 1C and 4a_ZnO NCs are coated mostly by amino acids such as lysine (Lys), methionine (Met), alanine (Ala), glutamic/aspartic acid (Glu/Asp) or asparagine/glutamine (Asn/Gln). It is in a good agreement with the coordination chemistry of zinc which binds preferably to the nitrogen ligands^110^. The previous works^13,101^ of our research group have confirmed the binding of zinc to the deprotonated carboxyl groups of aspartic/glutamic acid and gave an insight into the bio-ZnO NCs formation mechanism. We have also studied the interaction between zinc and the bacterial active functional with the use of capillary electrophoresis (CE)^111,112^. Therefore, it was revealed that the main groups involved in this kind of interaction are deprotonated carboxyl and amide groups derived from bacterial proteins and metabolites. This hypothesis was also supported by the computed IR spectrum of modeled Zn^2+^ complexes^111^. In the case of whole bacterial cells, the relationship between zinc and active groups support the aggregation process. However, in the case of use only bacterial supernatant containing bacterial metabolites and enzymes, it is possible to get a specific coating supporting the better colloidal dispersion (as shown in presented study). Based on the LDI-TOF-MS spectrometric results it can be concluded that amino acids detected as a bio-ZnO NCs surface deposit might be able to form some covalent or coordination bonds with nanoparticles surface. In the work of the Hematian et al.^113^ density-functional tight-binding (DFTB) calculations pointed out the formation of the covalent bonds between the amino acids and ZnO surface. Indubala with colleagues^114^ have prepared the ZnO nanorods capped with an alanine (Ala) amino acid—the molecular modeling results showed that the capping of nano-ZnO by alanine occurred via coordination bonding between zinc of ZnO and oxygen of the carboxylic group of the Ala amino acid. Moreover, Molnár et al.^115^ have proved that the coating of Au NCs through proteins occurred through the Van der Waals forces mainly between the nitrogen and sulphur atoms of the proteins and the surface of NCs. Intriguingly, Hematian et al.^113^ have also underlined the relationship between BSA amino acids interaction with ZnO and the crystallographic surfaces of zinc oxide. As mentioned before, the ZnO {0001} surfaces are found to be polar while the {1010} and {1120} surfaces are non-polar^32,33^. The DFBT simulations shown in the^113^ pointed out the strongest binding between specific amino acids and {1010} ZnO surfaces. Then, such a deep crystallographic and molecular study might be helpful in better understanding of nanoparticles-protein interaction and natural coating process.

In the case of extracellularly synthetized 1C and 4a_ZnO NCs, the coating process might occur through both, covalent for the asparagine (Asp), glutamine (Glu), glycine (Gln), lysine (Lys), serine (Ser) and coordination bonding for the alanine. Also the binding through the sulfur for the methionine (Met) possibly may take place. Nevertheless, the more detailed molecular study is require to confirm this statement.

To sum up, based on the all obtained experimental data as well as on our previous papers^10,13,116^ we would possible mechanism of bio-ZnO NCs formation during the extracellular synthesis can be proposed.

Our proposed mechanism can be represented by the overall reaction:

$$\begin{gathered} \left[ {{\text{Zn}}\left( {{\text{H}}_{{2}} {\text{O}}} \right)_{{6}} } \right]^{{{2} + }} + \left( { - {\text{COO}} - {\text{groups}}\;{\text{of}}\;{\text{bacterial}}\;{\text{metabolites}}} \right) \to \left[ {{\text{Zn}}\left( {{\text{OH}}} \right)\left( {{\text{H}}_{{2}} {\text{O}}} \right)_{{5}} } \right]^{ + } \hfill \\ \to \left[ {{\text{Zn}}\left( {{\text{OH}}} \right)_{{4}} } \right]^{{{2} - }} \to {\text{Zn}}\left( {{\text{OH}}} \right)_{{2}} + {\text{ 2OH}} - \to {\text{ZnO NCs }} + {\text{ H}}_{{2}} {\text{O}} \hfill \\ \end{gathered}$$

During all the process we have observed similar tendency about the pH—it was increased from 5.7 (the pH of medium before the inoculation) to around pH = 8 at the end of the biosynthesis. It clearly confirms the major role of deprotonated carboxyl groups of amino acids such as glutamic/aspartic acid (COO^–^; pKa 2–4) groups in the Zn^2+^_(aq)_-protein binding process and the subsequent [Zn(OH)_4_]^2−^ formation. The final step of reaction includes the alkali decomposition of intermediates to the zinc oxide. Then, taking into consideration obtained FT-IR results as well as the MALDI-TOF-MS we can assumed that one of the major group produced by probiotic bacteria are metabolites containing the deprotonated carboxyl groups such as aspartic or glutamic acid of peptides. Moreover, the mass spectrometry analysis verified the type of amino acid interacting with the zinc ions and the presence of water molecules confirmed the presence of zinc aquacomplexes. However, a further and more detailed study regarding the specific bacterial metabolites produced during biosynthesis is necessary.

## Patents

The details of both isolation method as well as nanocomposites synthesis method using *Latilactobacillus curvatus*, and *Limosilactobacillus fermentum* mentioned in this study is covered by a patent application in Poland – no. P.441585.

## Conclusions

Due to the growing interest in ZnO NCs biological production, the seeking for new synthesis sources as well the comprehensive and deep physicochemical characterization of bio-nanoparticles is required. In presented work, for the first time, the interdisciplinary approach including the broad range of instrumental techniques were applied to compare the chemically and extracellularly synthetized ZnO NCs. Furthermore, two probiotic strains isolated from milk, *Lactobacillus fermentum* (4a) and *Lactobacillus curvatus* (1C), were found to be effective, environmentally friendly and rich in specific naturals bioactive compounds source for bio-ZnO NCs formation. The use of microscopic, spectroscopic, spectrometric methods as well as the NCs stability analysis confirmed the existence of organic coating around the 1C and 4a_ZnO NCs nanoparticles core. The more detailed analysis of surface organic deposit revealed that it consist of mostly amino acids such as glutamic/aspartic acid, glutamine/asparagine, serine, methionine and others. The instrumental analysis confirmed the strong dependence of NCs concentration on their colloidal stability, photoluminescence and presence of organic deposit. Bio-ZnO NCs were found to be more stable during 7 days of investigation, compared to the chemical one. The highest (1000 µg/mL) concentration of all tested samples resulted in some suppression of PL signals which is associated with the colloidal stability as well as the NCs coating. The presence of organic deposit influence also the nanoparticles photocatalytic properties—for both types of biologically obtained ZnO NCs the degradation of methylene blue (MB) was lower compared to chemical and uncoated nanoparticles. Based on the obtained data, it is very difficult to make a clear judgment about which type of nanoparticles is “better”. Both types of synthesis provide some advantages—CHEM_ZnO NCs were found to be better photocatalyst, but were less stable than 1C and 4a_ZnO NCs. On the other hand, biological approach is definitively simpler and more environmentally friendly than chemical. Moreover, bio-ZnO NCs provide good biological properties (i.e. antimicrobial) additionally enhanced by natural origin surface coating. Then, the broad physicochemical characterization seem to be crucial step giving a deep insight into tested ZnO NCs properties and might be a fundament for the further discussion about nanoparticles application. In summary, the applied interdisciplinary approach allowed to assumption that the bio-ZnO NCs formation is strongly related with the bacterial metabolites containing the deprotonated carboxyl groups (mainly aspartic/glutamic acid). During the whole process, the increase of pH was observed—the start conditions included the pH around 5.7 where the zinc ions creates the stable aquacomplexes ([Zn(H_2_O)_6_]^2+^). Due to the ability of zinc aquacomplexes to exchange their water molecules with specific ligands, the interaction between Zn^2+^_(aq)_ and bacterial metabolites have occurred. As a result of such an interaction and the increment of pH (to value around 8), the alkali decomposition of intermediates took place. In the consequence, the ZnO NCs are formed.

## Data Availability

The datasets generated and/or analyzed during the current study are not publicly available due to the fact that the results/methods reported in present study are covered by a patent application in Poland – no. P.441585 but are available from the corresponding author on reasonable request.
